# No evidence of SARS-CoV-2 reverse transcription and integration as the origin of chimeric transcripts in patient tissues

**DOI:** 10.1073/pnas.2109066118

**Published:** 2021-08-03

**Authors:** Rhys Parry, Robert J. Gifford, Spyros Lytras, Stuart C. Ray, Lachlan J. M. Coin

**Affiliations:** ^a^School of Chemistry and Molecular Biosciences, University of Queensland, Brisbane, QLD 4072, Australia;; ^b^Medical Research Council–University of Glasgow Centre for Virus Research, Glasgow G61 1QH, United Kingdom;; ^c^Division of Infectious Diseases, Department of Medicine, Johns Hopkins University School of Medicine, Baltimore, MD 21205;; ^d^Department of Microbiology and Immunology, University of Melbourne at The Peter Doherty Institute for Infection and Immunity, Melbourne, VIC 3000, Australia;; ^e^Department of Clinical Pathology, University of Melbourne, Melbourne, VIC 3000, Australia;; ^f^Department of Infectious Disease, Imperial College London, London SW7 2AZ, United Kingdom;; ^g^Institute for Molecular Bioscience, University of Queensland, Brisbane, QLD 4072, Australia

There is interest in understanding the mechanisms that underlie reports that patients infected with severe acute respiratory syndrome coronavirus 2 (SARS-CoV-2) remain PCR positive many weeks after initial infection. The recent paper by Zhang et al. (1) suggests a potential explanation of this phenomenon by claiming that SARS-CoV-2 RNA can integrate into the genome of infected human cells. The authors also reanalyze RNA sequencing (RNA-seq) data and report that SARS-CoV-2−host chimeric reads are present in cells and patient tissues. Given the potential implications of this research on the long-term impacts of COVID-19, we feel that it’s necessary to scrutinize the evidence presented.

To determine whether SARS-CoV-2 RNA might be retrotranscribed and integrated into the genome, the authors conducted a proof-of-principle experiment where human lung cells (Calu3) and kidney cells overexpressing class I transposable elements and wild type (HEK293T-L1/HEK293T) were infected with SARS-CoV-2 and subjected to high-throughput DNA sequencing (1).

The very low frequency of identified chimeric events (Table 1) suggests that SARS-CoV-2 integration into the host genome is unlikely. Given that the HEK293T-L1 model increases detection of “rare integration events” and L1 can retrotranspose any polyadenylated cellular RNA (2) and “insertions” are found preferentially in protein-coding exons, a bias unknown to L1 endonuclease insertions (3), these findings are likely spurious. Additionally, 2 of the identified 61 chimeric nanopore genomic DNA (gDNA) reads contain human DNA from separate chromosomes (chr1,chr22 and chr18,chrX, respectively), suggesting a portion of chimeric gDNA nanopore reads have arisen due to infrequent technical artifacts, such as base-calling software not recognizing an open-pore state between distinct molecules.

**Table 1.** Summary of gDNA sequencing and chimeric reads identified

Accession	Cell line	Sequencing strategy	Chimeric reads/total reads	Percent of library
SRR14289057	HEK293T	Illumina Enrichment	2/71,263,270	0.000003
SRR14216062	Calu3	Illumina Enrichment	3/94,274,616	0.000003
SRR14216061	Calu3	Illumina Enrichment	2/103,608,699	0.000002
SRR14163829	HEK293T-L1	Nanopore	61/12,053,919	0.0005
SRR14136237	HEK293T-L1	Illumina	8/109,004,386	0.000007
SRR14136236	HEK293T-L1	Illumina	9/178,953,858	0.000005

Further to this, the authors reanalyzed published sequencing data and identified SARS-CoV-2 and human host chimeric reads in vitro and in patient RNA-seq (1). They show that the fraction of human–viral chimeric reads derived from negative-sense SARS-CoV-2 RNAs is higher in patients than that observed in vitro. These data are presented as evidence of SARS-CoV-2 integration and transcription. We question the presented data as evidence for this phenomenon for several key reasons. Firstly, chimeric virus−host reads are often reported in RNA-seq (4), including SARS-CoV-2 (5). This is partly due to complementary DNA fusions introduced during the reverse transcription step of library preparation (6). Secondly, larger pools of negative-sense SARS-CoV-2 chimeric reads may be due to differences in RNA extraction, library preparation, and sequencing platform. Given that negative-sense RNA is formed during the replication of SARS-CoV-2 and a template for messenger RNA production, significant variation of negative-sense reads is expected in patient RNA samples. Finally, there is no evidence of coronaviruses ever having integrated into the germline of host species, as might be expected if retrotranscription and integration occurs in nature, as systematic screening of >750 animal species failed to identify any coronavirus-derived endogenous viral elements (7).

Given the inappropriate interpretation of high-throughput sequencing methods and improper experimental design by the authors (1), we ask for restraint about the conclusions presented by the study. It remains unlikely that retrotranscription and integration of the SARS-CoV-2 genome in patients happens at any notable frequency, or even at all.
